# Vaccinations Do Not Increase Arthritis Flares in Juvenile Idiopathic Arthritis: A Study of the Relationship between Routine Childhood Vaccinations on the Australian Immunisation Schedule and Arthritis Activity in Children with Juvenile Idiopathic Arthritis

**DOI:** 10.1155/2020/1078914

**Published:** 2020-08-04

**Authors:** Naba M. Alfayadh, Peter J. Gowdie, Jonathan D. Akikusa, Mee Lee Easton, Jim P. Buttery

**Affiliations:** ^1^Department of Paediatrics, Monash University, Melbourne, Victoria, Australia; ^2^Departments of General Paediatrics and Paediatric Rheumatology, Monash Children's Hospital, Clayton, Melbourne, Victoria, Australia; ^3^Rheumatology Service, Department of General Medicine, Royal Children's Hospital, Melbourne, Victoria, Australia; ^4^Murdoch Children's Research Institute, Melbourne, Victoria, Australia; ^5^Department of Paediatrics, The University of Melbourne, Melbourne, Victoria, Australia; ^6^Surveillance of Adverse Events following Vaccination in the Community (SAEFVIC), Murdoch Children's Research Institute, Melbourne, Victoria, Australia; ^7^Monash Immunisation, Monash Health, Clayton, Victoria, Australia; ^8^Infection and Immunity, Monash Children's Hospital, Monash Health, Clayton, Victoria, Australia; ^9^Monash Centre for Health Research and Implementation, Monash University, Clayton, Victoria, Australia

## Abstract

**Background:**

Juvenile idiopathic arthritis (JIA) is a collective term for a group of inflammatory conditions of uncertain origin, which causes chronic arthritis in one or more joints. The clinical course of JIA is characterised by episodes of increased activity, termed flares. Vaccinations have previously been proposed as a “trigger” for some flares, although evidence supporting this is scant.

**Objective:**

To explore whether routine childhood vaccinations are associated with an increased risk of flares of arthritis activity in children with JIA.

**Methods:**

Patients aged below 6 years with a diagnosis of JIA were recruited from the Rheumatology Clinical Database at the Royal Children's Hospital, Melbourne, Australia, from 1 January 2010 to 30 April 2016. Patient immunisation status was cross-checked with the Australian Childhood Immunisation Register (ACIR). The self-controlled case series methodology (Rowhani-Rahbar et al., 2012) was applied to determine whether the risk of arthritis flares in the three months following immunisation was greater than the baseline risk for each patient.

**Results:**

138 patients were included in the study. 32 arthritis flares occurred in the 90 days following immunisation. The risk of arthritis flares during the 90 days following immunisation was reduced compared with patients' baseline risk (RR 0.59 (95% CI 0.39-0.89, *p* = 0.012)).

**Conclusion:**

Routine childhood immunisations were not associated with arthritis flare onset in patients with JIA. The risk of arthritis flares in the 90 days following vaccination was lower than the baseline risk. In the context of COVID19, vaccination will not increase interaction with the healthcare system beyond the immunisation encounter.

## 1. Introduction

Juvenile idiopathic arthritis (JIA) is the term used to describe a group of autoimmune inflammatory rheumatologic conditions in childhood whose hallmark feature is chronic arthritis [1–3]. Affecting 1 in 1000 Australian children [1, 2, 4, 5], JIA is characterised by a relapsing and remitting course with periods of complete quiescence, followed by periods of active synovitis, called flares [1]. While the triggers for most flares remain uncertain, they may be precipitated by viral illnesses or changes in medication [1–3].

Vaccination initiatives prevent millions of deaths annually through the prevention of communicable diseases [6–9]. However, in children with immunological conditions, such as JIA, the safety of vaccinations has been questioned by parents and healthcare providers [10–12]. Concerns regarding vaccination possibly initiating flares of disease affect clinician behaviour in immunising children with JIA. Independent surveys in Britain and Brazil have demonstrated that clinician uncertainty and concern about the contribution of vaccinations to flares resulted in discordance in vaccination practice [10, 11]. Some delayed vaccination until certain criteria, such as being well for a certain period, were met, while others vaccinated regardless [11, 12].

A variety of studies have tried to address this issue previously. 17 of the 24 studies identified in the available literature were conducted in a European setting [13–38], and most studies were small, with only four analysing over 100 children [16, 21, 23, 36]. There was one randomised controlled study [23], addressing the safety of the combined measles-mumps-rubella (MMR) vaccine. Many studies included flare activity as a secondary outcome, with flare activity assessed by parent interview 3-6 months post vaccination. Four routine vaccinations have not previously been assessed in children with JIA: diphtheria-tetanus-pertussis (DTP), *Haemophilus influenzae* type B (Hib), inactivated polio vaccine (IPV), and the rotavirus vaccine [13–21, 23–38]. As the onset of JIA is typically after the first year of life, infant immunisations are less studied.

We sought to examine whether there was a temporal correlation between routine vaccination in early childhood and flares of arthritis in an Australian cohort of patients with JIA to help inform vaccination recommendations for healthcare providers and patients.

## 2. Methods

A retrospective cohort study was performed using the Rheumatology Database at the Royal Children's Hospital (RCH) in Melbourne, Australia. This database is a rheumatology-specific clinical tool used in the inpatient and outpatient context to document patient clinical encounters. Clinical information from medical staff, specialist rheumatology nursing staff, parent phone calls, and general practitioner phone calls are entered at the time of the clinical encounter for all patients. Data was extracted and reviewed for all patients seen between 1 January 2010 and the 30 April 2016. Additional dates of vaccine administration were accessed from the Australian Childhood Immunisation Register (ACIR), a centralised database which enables registered vaccine providers to record vaccination administration information for Australian children. Patient demographics, disease activity at time of review or contact, and immunisation data were then collected for analysis.

All patients in the database under the age of 6 years who had a diagnosis of JIA and who were seen during the study period were potentially eligible for inclusion. The other inclusion criteria were as follows: (a) received an immunisation during the study period; (b) had four or more rheumatology consultations, to provide a sufficient baseline for the degree of flare propensity for each patient; and (c) had complete ACIR immunisation data for the period studied. Children over the age of 6 years were not included as accurate immunisation data for this population was not available.

Disease duration was calculated from diagnosis to the end of the control period in the study. The first visit to the Rheumatology Clinic at the Royal Children's Hospital was used as a proxy for the date of diagnosis, and the time of a child's 7^th^ birthday was used as the proxy for the end of the control period.

Medication exposure for children at the time of vaccination was assessed by evaluating the medical visits 6 months prior to vaccination and recording the medication plan for the time leading up to vaccination. As some children had more than one vaccination analysed in our study, medication exposure was assessed through the number of vaccination episodes, rather than the number of children. For the purposes of the study, it was assumed that all study participants adhered to the plan outlined by their physician.

An arthritis flare (in either the post vaccination risk interval or the control periods) was defined as specific a mention of “flare” in clinician notes, a documented increase in medication dose, or a documented increase in joint count.

It was anticipated that the exact timing of flare onset in JIA can be difficult to establish and that dates of flare onset entered on the database would be approximate. As such, a standardised approach was taken to determine the date of onset of flares to ensure consistency in data interpretation (Appendix A).

### 2.1. Patient Consent

This study was exempted from obtaining individual patient consent due to it being a retrospective clinical audit of vaccination administration and arthritis flares.

### 2.2. Ethics

This study received ethics approval from the Royal Children's Hospital's (RCH) Human Research Ethics Committee (36085A) and from Monash University's Human Research Ethics Committee (CF16/1818–2016000931).

### 2.3. Statistical Analysis

The self-controlled case series (SCCS) methodology is an accepted pharmacovigilance technique for analysing adverse events following discrete interventions, including immunisations [39–41]. The SCCS was designed for the analysis of the temporal association between a time-dependent exposure, such as medication or immunisation and an outcome [40, 41].

**Figure 1:**
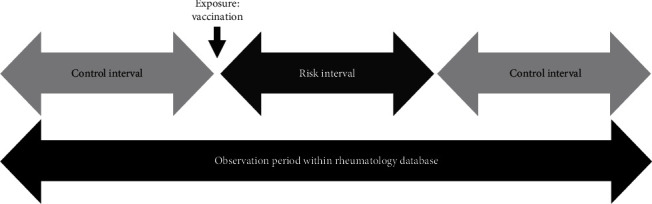
Intervals of analysis in the self-controlled case series methodology.

The SCCS methodology divides the study period into the risk interval and the control interval (Figure 1). The risk interval is the period following the administration of a vaccine in which the risk of a flare is expected to be impacted by the exposure. The risk interval selected was three months (90 days) following vaccination. The control periods were the periods during which the child was a participant in the Rheumatology Database before vaccination and after the risk interval [42]. The duration of the control interval varied between a few months and up to 4 years for the studied population. The rate of flares per day observed was calculated for each patient in their risk and control periods.

Two sensitivity analyses were performed to evaluate the findings of this study. The first excluded any uncertain flare dates by censoring all patients for whom flare dates were not accurately recorded by the clinician at the time of the clinical encounter and for whom the standardised data entry approach recorded the visit date as the flare date. The second sensitivity analysis reduced the risk window to 42 days and included only patients with certain flare onset dates.

## 3. Results

One hundred and thirty-eight children with JIA were included; 70% were female. The median age at first visit was 3.2 years (range 1.1-6.0), and the median disease duration was 3.8 years from diagnosis. The JIA subtype and gender distribution are summarised in Table 1. The majority (*n* = 79, 57.3%) had oligoarticular JIA. No child had rheumatoid factor positive polyarticular JIA.

60% of the children included were on treatment with immunosuppressive therapy in the 6 months prior to vaccination. Therapy included methotrexate (22%), NSAIDS - mainly naproxen (21%), intra-articular steroid injections (9%), oral prednisolone (4%), adalimumab (3%), and etanercept (1%).

During the 90 days following immunisation, children were less likely to experience arthritis flares than during the control interval. The relative risk of arthritis flares occurring in the postvaccination window was 0.59 (95% CI 0.39-0.89). The sensitivity analyses were consistent with the primary analysis (Table 2).

## 4. Discussion

This study has further confirmed the safety of routine childhood vaccinations in children with JIA. Our study design enabled us to study all vaccinations administered to children beyond 12 months of age with JIA across their early childhood. These include, for the first time, DTP, Hib, and the inactivated poliovirus vaccination.

Previous studies each analysed 3 vaccinations or less, with 21 studies analysing a single vaccine. Our study is the first to look at how the Australian National Immunisation Program schedule in its entirety affects children. These results offer strong support for the vaccination of children aged under 6 years with JIA. The Australian Immunisation Schedule is comprehensive, containing vaccine antigens shared with many international programs.

Our results augment previous studies, most of which found no increased risk of flare following vaccination [13–38]. Of the 24 studies identified in our literature review, only one case report attributed a flare in an 11-year-old female patient with systemic JIA to monovalent rubella vaccine [37]. She experienced a severe relapse of her disease, including congestive heart failure, five days after vaccination. The authors hypothesised that the association between the vaccination and this severe flare could have been due to the molecular mimicry between rubella and JIA. While monovalent rubella vaccine is not used in Australia, MMR vaccine (utilising the Wistar RA 27/3 rubella strain) was included, with 61 doses examined.

Our study is the first to employ the SCCS methodology to analyse flares in children with JIA postvaccination. The SCCS was developed for the analysis of the temporal association between a time-dependent exposure, such as a medication or immunisation, and an outcome [40, 41]. “Self-controlled” denotes that patients act as their own controls. This offers the advantage of negating the impact of most confounders, except for time, age, and seasonality. It controls for social status, household income, genetic risks, and many other implicit confounders [39–41].

The “risk window” following vaccination when the rate of arthritis flares may potentially be increased has not been agreed upon. The risk intervals used in studies using the SCCS methodology rely largely on biological plausibility, expert opinion, and precedents in the literature. Risk windows described for potential autoimmune adverse events following immunisation typically vary between 6 weeks and 3 months. We selected 90 days (3 months) as the risk interval for our primary analysis, and six weeks was used for the sensitivity analysis, with results consistent in both analyses [41, 43].

The reduced risk of arthritis flares postvaccinations observed in our findings may be due to several factors. Children may be generally “healthier than normal” at the time of vaccination (the “healthy vaccinee effect”), as parents and providers may delay vaccination until children are free of viral infections and have been generally well for a period of time. Secondly, every vaccination represents an interaction with a healthcare provider who may have initiated an intervention or changed a child's medications, resulting in improved health in the 90 days following vaccination.

Potential limitations of our study include the use of the clinical Rheumatology Database for retrospective research, which includes subjective free-text clinical notes, and the inability to examine beyond 6 years of age due to the lack of information in the ACIR after this age. To mitigate these risks, we utilised the inclusion criteria requiring patients to have sufficient data on the ACIR & Rheumatology Databases and created standardised Rheumatology Database extraction and study data entry rules (Appendix B). On 30 September 2016, Australia introduced an all-age immunisation database, the Australian Immunisation Register, an extension of the ACIR [43]. As the AIR accrues more immunisation status information for older children with JIA, the assessment of adolescent-administered vaccinations, such as the human papilloma vaccine, will also be feasible [43]. Finally, due to a lack of reliable ascertainment within the database, the main limitation of our study was the inability to include uveitis incidence as part of our definition of “flare.” Thus, our study only analysed arthritis flares in JIA.

The flare risk following rotavirus vaccination, which is only administered in infancy, could not be analysed in our study. The youngest child who satisfied the inclusion criteria was 12 months of age, consistent with the mean age of JIA diagnosis [1, 8].

It is also possible that changes in therapy in the period before immunisation may have altered the risk of flare following immunisation for individual patients. Immunisation administration was independent of Rheumatology Clinic visits. Medications may have been increased if joints were active or decreased if joints were inactive. Discrete individual dosing changes were not systematically captured across the entire study period, and potential confounding related to medication changes remains a possibility. However, across the cohort, there is unlikely to have been an overall bias towards one outcome in the risk period.

## 5. Conclusion

In young children with JIA, routine childhood vaccinations do not increase the risk of arthritis flares.

These findings should encourage clinicians and families to vaccinate children even the setting of the COVID19 pandemic. Outside of the immunisation encounter, vaccination will not increase risk of flares or healthcare system interaction.

## Figures and Tables

**Table 1:** JIA patient demographics by subtype.

JIA subtype	Number (%)	Female (%)	Mean disease duration
Oligoarticular	66 (48.6)	49 (73%)	3.7
Oligoarticular extended	12 (8.7)	10 (83%)	3.2
Polyarticular rheumatoid factor negative	27 (19.6%)	20 (74%)	3.6
Systemic JIA	8 (5.8%)	5 (63%)	3.1
Psoriatic	5 (3.6%)	1 (20%)	3.3
Enthesitis-related arthritis	1 (0.72%)	0 (0%)	1.8
Undifferentiated	19 (13.8%)	12 (63%)	3.9
Total	138	97	

**Table 2:** Self-controlled case series analyses.

Analysis	RR (95% CI)	*p* value	Patients (*n*)	Arthritis flares (*n*)
Primary analysis risk interval: 90 days	0.59 (95% CI 0.39-0.89)	0.012	138	32
Sensitivity analysis 1; RI: 90 days, excluding nonspecific flare dates	0.38 (95% CI 0.21-0.67)	0.009	122	16
Sensitivity analysis 2; RI: 42 days, excluding nonspecific flare dates	0.12 (95% CI 0.02-0.49)	0.003	122	2

**Table 3:** National Immunisation Schedule for Australian children, organised by the ages at which the immunisations are recommended [7].

Age	Vaccine
Birth	Hep B
2 months	DTP-IPV-Hep B-Hib, PCV, RV5
4 months	DTP-IPV-Hep B-Hib, PCV, RV5
6 months	DTP-IPV-Hep B-Hib, PCV, RV5
12 months	Hib-Men C, MMR
18 months	VZV—until 2013 2013 onward—MMRV, DTP
4 years	DTP-IPV Until 2013—MMR

Hep B: hepatitis B; DTP: diphtheria-tetanus-pertussis; IPV: inactivated poliomyelitis vaccine; Hib: *Haemophilus influenzae* type B; PCV: pneumococcal conjugate vaccine; RV: rotavirus; Men C: meningococcal C; MMR: measles-mumps-rubella; VZV: varicella zoster.

## Data Availability

The dataset used and analysed for this study is available from the Rheumatology Database at the Royal Children's Hospital and can only be made available through formal application to the Department of Rheumatology.

## Appendix

### A. Standardised Rules for Entering Patient Flare Data into the Electronic Database

1. If only the week during which the flare occurred is stated, the Monday of that week was taken as the flare date for analysis
2. If only the month during which the flare occurred is stated, the 15^th^ day of that month was taken as the flare date for analysis
3. If it is stated that the flare occurred at the “start” of the month, the first day of that month was taken as the flare date for analysis
4. If it is stated that the flare occurred at the “end” of the month, the last day of the month was taken as the flare date for analysis
5. If it is stated that the flare began “three to four weeks ago,” the flare date taken for analysis purposes was the midpoint between the two stated dates
6. If it is stated that the flare occurred when the patient was “*x*” years old, the patient's birthday with “*x*” years added was taken as the flare date for analysis
7. If the flare date was not stated but a flare was clearly occurring during the visit, the visit date was taken as the flare date for analysis. However, these dates were noted and were excluded in the sensitivity analysis
8. If the patient was not in complete remission and another joint became involved, this was taken to be part of the same flare

### B. Immunisation Schedule and Developments during the Study Period

The Australian Immunisation Schedule is detailed in Table 3.

#### B.1. Changes to Immunisation Schedule during Study Period

Several changes were applied to the above immunisation schedule during the study period. In 2013, the pneumococcal conjugate vaccine (PCV) changed from 7-valent (PCV7) to 13-valent (PCV13). In infancy, diphtheria-tetanus-pertussis (DTP), hepatitis B (Hep B), *Haemophilus influenzae* type b (Hib), and inactivated poliomyelitis (IPV) were administered as a combination hexavalent vaccine at 2, 4, and 6 months. At 12 months, the combination of Hib and Group C meningococcal conjugate vaccine was used, in conjunction with measles-mumps-rubella (MMR) vaccine dose 1 with varicella vaccine administered at 18 months of age. Dose 2 MMR was scheduled at 3.5-4 years of age with DTP-IPV combination vaccine until 2013. From 2013, dose 2 was scheduled as MMR-varicella (MMRV) at 18 months of age with an additional dose of DTP vaccine at that time. Annual trivalent inactivated influenza vaccine (TIV) or quadrivalent influenza vaccine (QIV) from 2015 was funded and recommended for children with chronic diseases including rheumatologic disease.

The oral rotavirus vaccine used in Victoria was the oral live attenuated pentavalent bovine-human reassortant vaccine (RV5) until 1 July 2017, when the schedule changed to the oral human monovalent G1P [8] (RV1) vaccine.

## References

[1] Gowdie P. J., Tse S. M. L. (2012). Juvenile idiopathic arthritis. *Pediatric Clinics of North America*.

[2] Kulas D. T., Schanberg L. (2001). Juvenile idiopathic arthritis. *Current Opinion in Rheumatology*.

[3] Ravelli A., Martini A. (2007). Juvenile idiopathic arthritis. *The Lancet*.

[4] Prakken B., Albani S., Martini A. (2011). Juvenile idiopathic arthritis. *The Lancet*.

[5] Weiss J. E., Ilowite N. T. (2007). Juvenile idiopathic arthritis. *Rheumatic Disease Clinics of North America*.

[6] Ageing AGDoHa (2013). *Myths and Realities: Responding to Arguments against Vaccination, A Guide for Providers*.

[7] Crawford N. W., Buttery J. P. (2013). Adverse events following immunizations: fact and fiction. *Paediatrics and Child Health*.

[8] Program AGDoHIA (2016). Immunise Australia Program Australia: Department of Health. http://immunise.health.gov.au/internet/immunise/publishing.nsf/Content/introduction-ai.

[9] Schattner A. (2005). Consequence or coincidence?: The occurrence, pathogenesis and significance of autoimmune manifestations after viral vaccines. *Vaccine*.

[10] Conti F., Rezai S., Valesini G. (2008). Vaccination and autoimmune rheumatic diseases. *Autoimmunity Reviews*.

[11] Davies K., Woo P. (2002). Immunization in rheumatic diseases of childhood: an audit of the clinical practice of British Paediatric Rheumatology Group members and a review of the evidence. *Rheumatology*.

[12] Silva C. A. A., Terreri M. T. R. A., Aikawa N. E. (2010). Prática de vacinação em crianças com doenças reumáticas. *Revista Brasileira de Rheumatologia*.

[13] Aikawa N. E., França I. L. A., Ribeiro A. C., Sallum A. M. E., Bonfa E., Silva C. A. (2015). Short and long-term immunogenicity and safety following the 23-valent polysaccharide pneumococcal vaccine in juvenile idiopathic arthritis patients under conventional DMARDs with or without anti-TNF therapy. *Vaccine*.

[14] Borte S., Liebert U. G., Borte M., Sack U. (2009). Efficacy of measles, mumps and rubella revaccination in children with juvenile idiopathic arthritis treated with methotrexate and etanercept. *Rheumatology*.

[15] Camacho-Lovillo M. S., Bulnes-Ramos A., Goycochea-Valdivia W. (2017). Immunogenicity and safety of influenza vaccination in patients with juvenile idiopathic arthritis on biological therapy using the microneutralization assay. *Pediatric Rheumatology*.

[16] Carvalho L. M., de Paula F. E., Silvestre R. V. D. (2013). Prospective surveillance study of acute respiratory infections, influenza-like illness and seasonal influenza vaccine in a cohort of juvenile idiopathic arthritis patients. *Pediatric Rheumatology*.

[17] Dell’Era L., Corona F., Daleno C., Scala A., Principi N., Esposito S. (2012). Immunogenicity, safety and tolerability of MF59-adjuvanted seasonal influenza vaccine in children with juvenile idiopathic arthritis. *Vaccine*.

[18] Esposito S., Corona F., Barzon L. (2014). Immunogenicity, safety and tolerability of a bivalent human papillomavirus vaccine in adolescents with juvenile idiopathic arthritis. *Expert Review of Vaccines*.

[19] Farmaki E., Kanakoudi-Tsakalidou F., Spoulou V. (2010). The effect of anti-TNF treatment on the immunogenicity and safety of the 7-valent conjugate pneumococcal vaccine in children with juvenile idiopathic arthritis. *Vaccine*.

[20] Groot N., Pileggi G., Sandoval C. B. (2017). Varicella vaccination elicits a humoral and cellular response in children with rheumatic diseases using immune suppressive treatment. *Vaccine*.

[21] Heijstek M. W., Kamphuis S., Armbrust W. (2013). Effects of the Live Attenuated Measles-Mumps-Rubella Booster Vaccination on Disease Activity in Patients With Juvenile Idiopathic Arthritis. *JAMA*.

[22] Heijstek M. W., Pileggi G. C. S., Zonneveld-Huijssoon E. (2007). Safety of measles, mumps and rubella vaccination in juvenile idiopathic arthritis. *Annals of the Rheumatic Diseases*.

[23] Heijstek M. W., Scherpenisse M., Groot N. (2014). Immunogenicity and safety of the bivalent HPV vaccine in female patients with juvenile idiopathic arthritis: a prospective controlled observational cohort study. *Annals of the Rheumatic Diseases*.

[24] Kanakoudi-Tsakalidou F., Trachana M., Pratsidou-Gertsi P., Tsitsami E., Kyriazopoulou-Dalaina V. (2001). Influenza vaccination in children with chronic rheumatic diseases and long-term immunosuppressive therapy. *Clinical and Experimental Rheumatology*.

[25] Kasapçopur Ö., Çullu F., Kamburoðlu-Goksel A. (2004). Hepatitis B vaccination in children with juvenile idiopathic arthritis. *Annals of the Rheumatic Diseases*.

[26] Malleson P., Tekano J. L., Scheifele D. W., Weber J. M. (1993). Influenza immunization in children with chronic arthritis: a prospective study. *The Journal of Rheumatology*.

[27] Maritsi D., Vougiouka O., Vartzelis G. (2014). The response to hepatitis a vaccine in children with JIA on immunosuppresive treatment. *Pediatric Rheumatology*.

[28] Maritsi D. N., Coffin S. E., Argyri I., Vartzelis G., Spyridis N., Tsolia M. N. (2017). Immunogenicity and safety of the inactivated hepatitis A vaccine in children with juvenile idiopathic arthritis on methotrexate treatment: a matched case-control study. *Clinical and experimental rheumatology*.

[29] Nerome Y., Akaike H., Nonaka Y. (2016). The safety and effectiveness of HBV vaccination in patients with juvenile idiopathic arthritis controlled by treatment. *Modern Rheumatology*.

[30] Ogimi C., Tanaka R., Saitoh A., Oh-Ishi T. (2011). Immunogenicity of influenza vaccine in children with pediatric rheumatic diseases receiving immunosuppressive agents. *The Pediatric Infectious Disease Journal*.

[31] Pileggi G. S., de Souza C. B. S., Ferriani V. P. L. (2010). Safety and immunogenicity of varicella vaccine in patients with juvenile rheumatic diseases receiving methotrexate and corticosteroids. *Arthritis Care & Research*.

[32] Sengler C., Niewerth M., Kallinich T. (2014). Survey about tolerance of the AS_03_-adjuvanted H1N1 influenza vaccine in children with rheumatic diseases. *Clinical Rheumatology*.

[33] Soloshenko M., Alexeeva E., Bzarova T. (2017). FRI0154 the tolerability of vaccination against pneumococcus in children with juvenile idiopathic arthritis. *Annals of the Rheumatic Diseases.*.

[34] Toplak N., Subelj V., Kveder T. (2012). Safety and efficacy of influenza vaccination in a prospective longitudinal study of 31 children with juvenile idiopathic arthritis. *Clinical and Experimental Rheumatology*.

[35] Zonneveld-Huijssoon E., Ronaghy A., Van Rossum M. A. J. (2007). Safety and efficacy of meningococcal c vaccination in juvenile idiopathic arthritis. *Arthritis and Rheumatism*.

[36] Korematsu S., Miyahara H., Kawano T. (2009). A relapse of systemic type juvenile idiopathic arthritis after a rubella vaccination in a patient during a long-term remission period. *Vaccine*.

[37] Singer N., Wagner-Weiner L., Nanda K., Robinson A., Spalding S., Bükülmez H. (2014). Immunization with quadrivalent HPV vaccine (GARDASIL®) appears safe and induces antibody response in JIA: an interim analysis. *Annals of the Rheumatic Diseases*.

[38] Farrington C. P., Nash J., Miller E. (1996). Case series analysis of adverse reactions to vaccines: a comparative evaluation. *American Journal of Epidemiology*.

[39] Farrington C. P., Whitaker H. J., Hocine M. N. (2009). Case series analysis for censored, perturbed, or curtailed post-event exposures. *Biostatistics*.

[40] Whitaker H. J., Hocine M. N., Farrington C. P. (2009). The methodology of self-controlled case series studies. *Statistical Methods in Medical Research*.

[41] Hull B. P., Deeks S. L., McIntyre P. B. (2009). The Australian Childhood Immunisation Register--A model for universal immunisation registers?. *Vaccine*.

[42] Rowhani-Rahbar A., Klein N. P., Dekker C. L. (2012). Biologically plausible and evidence-based risk intervals in immunization safety research. *Vaccine*.

[43] Hull B. P., Mclntyre P. B. (2000). Immunisation coverage reporting through the Australian Childhood Immunisation Register — an evaluation of the third-dose assumption. *Australian and New Zealand Journal of Public Health*.

